# Academic and Employment Preferences of Nursing Students at the University of Las Palmas of Gran Canaria: A Cross-Sectional Study

**DOI:** 10.3390/nursrep14040241

**Published:** 2024-11-01

**Authors:** Andrea Ramos-Ramos, Claudio Alberto Rodríguez-Suárez, Candelaria de la Merced Díaz-González, José Verdú-Soriano, Miriam Berenguer-Pérez, Héctor González-de la Torre

**Affiliations:** 1 Insular Maternal and Child University Hospital Complex of Gran Canaria, Canary Health Service, Avda Marítima del Sur, s/n, 35016 Las Palmas de Gran Canaria, Spain; aramram1@gobiernodecanarias.org; 2 Nursing Department, Faculty of Healthcare Science, University of Las Palmas de Gran Canaria (ULPGC), Edificio Ciencias de la Salud, C/Blas Cabrera Felipe, s/n, 35016 Las Palmas de Gran Canaria, Spain; candelaria.diazg@ulpgc.es; 3 Research Support Unit of Insular Maternal and Child University Hospital Complex of Gran Canaria, Canary Health Service, Avda Marítima del Sur, s/n, 35016 Las Palmas de Gran Canaria, Spain; 4 Department of Community Nursing, Preventive Medicine, Public Health and History of Science, Faculty of Health Sciences, University of Alicante (UA), 03690 Alicante, Spain; pepe.verdu@ua.es (J.V.-S.); miriam.berenguer@ua.es (M.B.-P.)

**Keywords:** nursing education, nursing students, profession selection, professional competence

## Abstract

Background/Objectives: It is well known that there are differences in the academic and employment preferences of nursing students once they have completed their undergraduate studies in nursing. These preferences are largely influenced by students’ affinity for certain thematic areas over others. Therefore, the objective of this study was to identify the employment and academic preferences of third- and fourth-year Nursing Degree students at the University of Las Palmas de Gran Canaria (ULPGC). Methods: A cross-sectional, descriptive observational study was conducted among nursing students across three campuses of the ULPGC (Canary Islands, Spain). An online questionnaire was used to collect various sociodemographic and academic variables, as well as preferences across ten thematic areas. Descriptive and bivariate inferential analyses were performed, along with a correlation analysis among the areas. Results: The areas of highest preference were “Emergency Nursing”, “General Nursing”, and “Family and Community Nursing”. The areas of lowest preference were “Other Areas (teaching, management, research)”, “Mental Health and Psychiatric Nursing”, and “Geriatric Nursing”. Three clusters of closely correlated areas were identified: cluster 1 (Intensive and Critical Care Nursing, Emergency and Emergency Nursing and Operating Theatre and Anaesthesia Nursing), cluster 2 (Obstetric-Gynaecological Nursing–Midwifery, Paediatric Nursing and Mental Health and Psychiatric Nursing) and cluster 3 (remaining areas). A significant proportion of students expressed intentions to pursue postgraduate studies. Conclusions: Strategies should be implemented to enhance students’ preferences in the areas of “Mental Health and Psychiatric Nursing” and “Geriatric Nursing”, which are areas where there is a high demand for nurses. It is also necessary to increase their interest in research, management, and teaching. This study was not registered.

## 1. Introduction

Nurses constitute the largest professional group within the Spanish Health Care System, as reported by the Spanish National Institute of Statistics (INE). In 2023, the number of registered nurses, including midwives, was 356,255, representing 35.5% of the total healthcare workforce. This was followed by the medical profession, which accounted for 301,684 professionals, or 30.9% of the workforce [1]. However, these figures do not cover national needs. At the global level, the International Council of Nurses (ICN) estimates that the future global demand for nurses may increase to 13 million [2].

Recently, the ICN has made an urgent appeal to the member countries of the Group of Twenty (G20) to request that immediate actions be taken in the face of a global scarcity of nursing professionals, a situation that is even considered a ‘global crisis’ [3]. In Spain, the ongoing emigration of nurses to countries that offer better job stability, higher economic incentives, and enhanced support for social, cultural, and professional development poses a significant challenge [4]. This migration not only represents a loss of investment in the training of these professionals but also contributes to a talent drain, with adverse implications for the nation’s healthcare system. Spanish nurses are highly regarded internationally; in 2023, 1473 nurses applied for certification to practise abroad, with Norway, the United States, and the United Kingdom being the most sought-after destinations [4]. The existing shortages, compounded by this migratory trend, risk undermining the stability and effectiveness of the healthcare infrastructures in Spain [5].

In Spain, nursing studies consist of the University Degree in Nursing (4 years), Postgraduate studies (1–2 years), and Doctoral programmes (4 years full-time or 6 years part-time) [6]. Nursing students must complete theoretical and practical content, and in the last instance, they must complete several clinical rotations in different healthcare settings. However, not all students are able to rotate in all clinical areas, so that some students are not in special contact with all nursing specialities. There is a national agency, ANECA, which evaluates and monitors the quality of nursing studies in all Spanish universities.

Once the degree has been completed, the new graduate may opt for one of the following alternatives, or a combination of several (in some cases): (a) continuing with postgraduate training; (b) preparation for the admission tests for specialized nursing training (Resident Internal Nurse—EIR); (c) teaching in private academies, professional training modules and universities as a part-time professor; (d) research activity; (e) clinical activity in different fields; and (f) continuing professional education. Among the nursing specialities (EIR) contemplated in Royal Decree (RD) 450/2005 [7] are the following: Obstetric-Gynaecological Nursing, Paediatric Nursing, Mental Health Nursing, Family and Community Nursing, Occupational Nursing and Geriatric Nursing. In addition, the RD provides for an additional speciality, Medical-Surgical Nursing; however, the latter is not currently developed. In Spain, the number of vacancies available in these specialties is limited, so that in many cases, nurses are unable to carry out their professional practice in the area of their choice. The Spanish Health Care System is more rigid compared to other countries, and there is little flexibility in the choice of jobs for nurses. This is another reason why many nurses emigrate abroad, as this allows them to work in more preferred areas of nursing.

The final year of the Nursing Degree has been identified as the most critical period for professional decision making, particularly concerning the selection of practice destinations within various specialties [8], as well as for solidifying engagement with the profession [9]. It has been reported that the most preferred areas include the emergency department, operating theatre, paediatrics, or intensive care [10]. Several studies have indicated that these preferences are influenced by the level of technological advancement associated with each specialty [10,11]. Consequently, areas perceived as ‘low-tech’, such as psychiatry and geriatrics, tend to be the least preferred among nursing students [10,11].

It is known that nursing students’ preferences may change at different stages of their education and in the long term during their professional career, although a certain percentage of students maintain stable preferences over time. For instance, Anyango et al. found that 63.8% of students in their study retained their initial preferences by the end of their nursing programme, indicating that their studies did not significantly alter their preconceived career plans [12]. However, the exposure to specific specialties and areas, whether through rotational practice placements in certain departments or units or as a result of the established curriculum, can influence changes in these preferences [8]. Consequently, limited contact with or experience in certain specialties may lead to a lack of interest in these areas or a perceived deficiency in the necessary skills, ultimately resulting in missed career opportunities and hindered progression within those specialties [13].

The research on the selection of specific clinical areas by nursing students has identified various predictors, including age, gender, nationality, and the university where the students are trained [10,14,15]. However, other significant factors influencing the professional decisions of future graduates have also been recognized, such as the clinical environment, individual characteristics, and educational experiences [12,16]. Considering these factors, it is essential for educational institutions to play an active role in ensuring that students engage in practical experiences across all clinical settings. This approach not only broadens students’ knowledge and facilitates the acquisition of diverse skills but also fosters positive attitudes toward a wide range of patient populations and supports the recruitment of future nurses into all specialties and clinical areas [17].

As mentioned above, nursing students have various training and employment options available to them upon graduation. The shortage of specialized professionals in certain areas can negatively impact the quality of healthcare services, leading to poorer health outcomes and lower levels of patient satisfaction [18,19]. Understanding the academic and occupational preferences of future nursing graduates is crucial for identifying, on one hand, the clinical areas that are most preferred and, therefore, more likely to be adequately staffed, and on the other hand, the areas that are less attractive to this group but are critically important to the health system. On the other hand, although numerous studies have investigated nursing students’ preferences for various specialties and areas of practice, the relationships between these areas have not been sufficiently explored by researchers. The present study aimed to address this knowledge gap by conducting a correlation analysis to examine the possible relationships between the preferred areas. This could help in the development of joint strategies for different areas that could perhaps be considered related.

Therefore, the main aim of this study was to identify the employment and academic preferences of students in the third and fourth year of the Nursing’s Degree at the University of Las Palmas de Gran Canaria (ULPGC). As a secondary aim, we explored the possible relationship between the areas of preference reported by the nursing students.

## 2. Materials and Methods

### 2.1. Study Design

This study employed a cross-sectional, descriptive, and observational design with an analytical component. The STROBE reporting guidelines for observational and epidemiological studies were followed (STrengthening the Reporting of OBservational studies in Epidemiology) [20] (Supplementary Material Table S1).

### 2.2. Study Population and Setting

The study population was the 3rd- and 4th-year students of the Nursing Degree enrolled during the academic year 2023/2024 at the ULPGC. The ULPGC is a public university located on the island of Gran Canaria (Canary Islands, Spain), but with campuses on three different islands (Gran Canaria, Lanzarote, and Fuerteventura). Each of these campuses offers a Nursing Degree programme. The inclusion criterion was being a 3rd- or 4th-year nursing student enrolled during the 2023–2024 academic year at any of the three campuses. The only exclusion criterion was being a mobility student in the 3rd or 4th year of the Nursing Degree during the 2023–2024 academic year, specifically those participating in the System of Exchange between University Centres in Spain (SICUE) or the Erasmus Programme.

### 2.3. Sample Size

The reference population to be studied was the 430 students (n = 430) enrolled, with 268 students from the Gran Canaria campus, 80 from the Lanzarote campus, and 82 from the Fuerteventura campus. Accepting an alpha risk of 0.05 (confidence of 95%) for a precision of ±0.05 units in a bilateral contrast, it was estimated that a random sample of 374 students was required, considering that the estimated standard deviation in the population was 1.36. This calculation was based on the maximum standard deviation achieved for preference scores in the study by Afonso-Machado and González-de la Torre [21]. It was estimated that there would be no losses. The sample calculation was performed using the online GRANMO calculator [22].

### 2.4. Variables to Study

The following variables were considered: age, gender, previous health work experience (yes/no), nationality (Spanish/non-Spanish), marital status (single/married/divorced), number of children, university location (Gran Canaria Campus/Fuerteventura Campus/Lanzarote Campus), academic year (3rd/4th year), intention to access the specialized nursing training—EIR (yes/no), intention to access postgraduate training programmes—Official Masters (yes/no), intention to access doctoral programmes (yes/no), and ten thematic areas of employment preference (Paediatric Nursing, Obstetric-Gynaecological Nursing–Midwifery, Mental Health and Psychiatric Nursing, Emergency Nursing, Operating Theatre and Anaesthesia Nursing, General Nursing, Intensive and Critical Care Nursing, Family and Community Nursing—Primary Care, Geriatric Nursing and Other areas). The operational definitions of these areas can be found in Supplementary Material Table S2.

### 2.5. Instrument and Data Collection System

An online questionnaire similar to the one used in the study carried out at the University of La Laguna in 2022 by Afonso-Machado and González-de la Torre in an equivalent population [21] was used as a data collection instrument. The questionnaire was divided into two parts. In the first part, the sociodemographic variables under study were collected, as well as the intentions of accessing the specialized nursing training programme, postgraduate training, or doctoral programmes. The second part assessed preferences across ten thematic areas, with each area rated on a Likert scale ranging from 1 to 5, where 1 indicated “Not at all desired” and 5 indicated “Very much desired”. Higher scores corresponded to a stronger preference for the respective thematic area.

A non-probabilistic convenience sample was drawn from the study population. Data collection was conducted online using the Google Form^®^ tool, with the questionnaire link disseminated via each student’s institutional email, ensuring exclusive access to the intended participants. The data collection period extended from 20 December 2023 to 29 February 2024, during which three reminder emails were sent.

### 2.6. Data Analysis

A descriptive analysis was performed, expressing qualitative variables as percentages and frequencies and quantitative variables as means and standard deviations. The percentage of agreement was calculated for each area, this being calculated as the number of students who scored 4 or 5 in a thematic area divided by the total number of students and multiplied by 100. A bivariate inferential analysis was carried out with the chi-squared test to establish whether there were differences with respect to the categorical variables between the students according to the three campuses to which they belonged.

For quantitative variables, the Kolmogorov–Smirnov test showed the asymmetry of the distribution of area scores, so the non-parametric Mann–Whitney U test was used for the comparison of means between two groups and the Kruskall–Wallis test was used for more than two groups. In the latter case, the Dwass-Steel-Critchlow-Fligne test was used for the post hoc contrast. For each association analyzed, the effect size was calculated: for the Mann–Whitney test, the effect size was measured as a biserial rank correlation, and for the Kruskall–Wallis test, the effect size was measured by Kelley’s squared Epsilon measure. Coefficients between 0.10 and 0.30 were considered a small effect size, between 0.30 and 0.50 a medium effect size, and greater than 0.50 a large effect size [23].

Finally, a correlation analysis was carried out between the different thematic areas using Spearman’s Rho coefficient, considering positive moderate correlations to be those with values of between 0.5 and 1, and negative moderate correlations to be those with values of between −0.5 and −1. The correlational structure was represented graphically by means of a Heatmap with clusters. To establish the number of clusters to be included in the Heatmap, an exploratory factor analysis (EFA) by using principal components, oblimin rotation, and the number of factors based on parallel analysis was carried out, establishing that the number of clusters to be considered was the same as the number of factors identified in the EFA. The sample size was considered adequate for the EFA based on the subject–variable ratio following the recommendation that there should be at least 10 subjects for each variable (a minimum of 100 subjects for 10 areas) [24].

Statistical significance was set at α ≤ 0.05 for this study. Data analysis was carried out using JAMOVI v2.5.3.

### 2.7. Ethical Considerations

This research was approved by the Research Ethics Committee (CEI/CEIm) of Hospital Dr. Negrín, Las Palmas (Code N° 2023-515-1). Additionally, authorization to conduct the study was obtained from the management of the Faculty of Health Sciences at the ULPGC. All the participating students received an informative email outlining the study’s objectives and providing access to the online data collection form. Participation in the study was implied by the students’ access to and completion of the questionnaire, thereby indicating their informed consent. The data collection forms were designed to be anonymous, with no names or identifying information recorded. All the databases were anonymized to ensure confidentiality.

## 3. Results

### 3.1. Descriptive Analysis of the Sample

Of the potential study population (430 students from the three campuses), 179 students agreed to participate (n = 179), representing a response rate of 41.62% and a missing response rate of 58.38%. No participants were excluded from the study. The participants had a mean age of 25.2 ± 8.91 years, with 83.2% (149) identifying as female, 16.2% (29) as male, and 0.6% (1) as non-binary. Regarding the distribution by campus, 64.8% (116) of the participants were from the Gran Canaria Campus, 14.5% (26) were from the Lanzarote Campus, and 20.7% (37) were from the Fuerteventura Campus. Table 1 shows the frequencies and percentages of the variables considered according to the campus to which the participants belonged. Statistically significant differences were found between the groups of sites for the variables of marital status (*p* = 0.010), previous health work experience (*p* = 0.030), and intention to carry out postgraduate training (*p* = 0.002). Statistically significant differences were also found for age (Table 1).

### 3.2. Analysis of Preferences for Thematic Areas

The thematic areas with the highest scores and therefore indicated by the students as having the highest preference were General Nursing (M = 2.70 [95%CI: 2.56–2.84]) and Emergency Nursing (M = 2.70 [95%CI: 2.54–2.87]). In contrast, the areas with the lowest preference were Other Areas (teaching, management, research) (M = 1.43 [95%CI: 1.24–1.64]) and Mental Health and Psychiatric Nursing (M = 1.78 [95%CI: 1.60–1.95]). Table 2 shows the means with their 95% confidence intervals, standard deviations of the scores obtained for each thematic area, as well as the floor and ceiling percentages and the percentage of agreement for all the thematic areas.

The possible associations of the variables collected with respect to the 10 areas were explored. Table 3 shows all the means and standard deviations of the groups for each of the areas, as well as the *p*-values and effect sizes obtained for each of the bivariate analysis (third vs. fourth year, Gender Female vs. Male, children Yes vs. No, previous health work experience Yes vs. No and Intention nursing speciality, intention postgraduate training or intention Doctoral programme Yes vs. No).

In this analysis, multiple statistically significant results were found. Thus, in relation to the gender variable, women prefer Paediatric Nursing (*p* = 0.020) and Obstetric-Gynaecological Nursing (*p* = 0.002), with a moderate effect size in the latter case, while men prefer Emergency Nursing (*p* = 0.032) and Other Areas (teaching, management, research) (*p* = 0.001), also with a moderate effect size in the latter case. For the analysis of the variable Intention to carry out a nursing speciality, those who responded affirmatively show more interest in Paediatric Nursing (*p* = 0.001) and Obstetric-Gynaecological Nursing (*p* = 0.001), with medium effect sizes in both cases, while those who responded negatively show a greater predilection for General Nursing (*p* = 0.009) and Intensive and Critical Care Nursing (*p* = 0.012), although in this case, the effect sizes were smaller. No statistically significant differences were found between the three sites for any of the areas considered (Supplementary Material Table S3). Statistically significant differences were found for Emergency Nursing (*p* = 0.045), Family and Community Nursing (*p* = 0.029), Geriatric Nursing (*p* = 0.001), and Other Areas (*p* = 0.031), with respect to the variable of marital status (Supplementary Material Table S4).

### 3.3. Correlation Results on the Preference of Thematic Areas

Regarding the study of correlations, a moderate positive correlation was found between Intensive and Critical Care Nursing and Emergency Nursing (r = 0.510, *p* ≤ 0.001). The strongest negative correlation was found between Intensive and Critical Care Nursing and Mental Health and Psychiatric Nursing (r= −0.258, *p* ≤ 0.001). The complete correlation matrix, as well as the *p*-values for each co-relation, can be found in Table 4.

**Figure 1:**
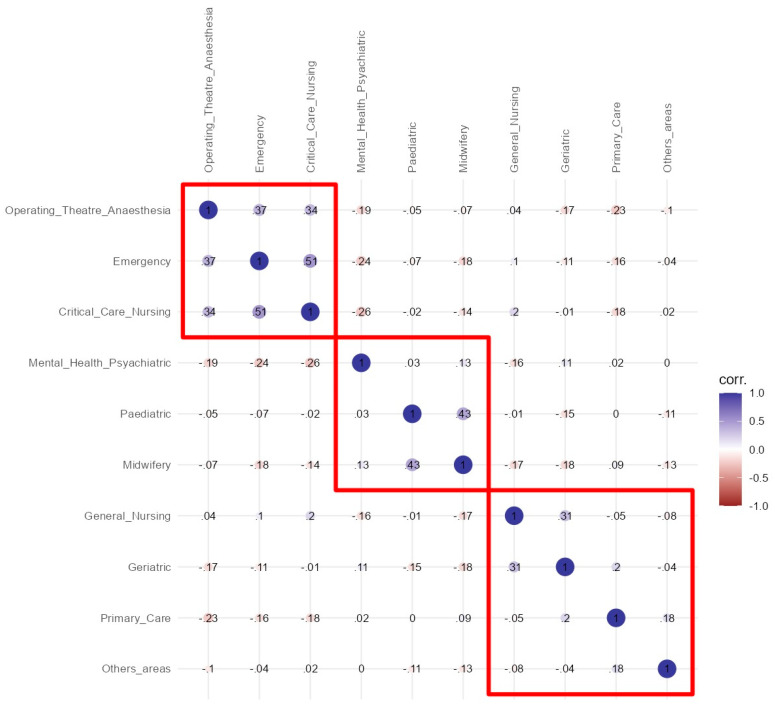
Heatmap of correlation structure.

Finally, the EFA indicated three factors, so a Heatmap was made based on the three clusters (Figure 1). In Figure 1, it can be seen how cluster 1 is composed of three areas (Intensive and Critical Care Nursing, Emergency Nursing and Operating Theatre and Anaesthesia Nursing), a second cluster that includes the areas of Obstetric-Gynaecological Nursing–Midwifery, Paediatric Nursing and Mental Health and Psychiatric Nursing and a third cluster composed of the remaining four areas. In this first cluster, the correlations between the three areas are in all cases positive, with r-values above 0.30.

## 4. Discussion

The aim of this study was to identify the academic and employment preferences of third- and fourth-year Nursing Degree students at the ULPGC. Regarding the gender distribution, the sample exhibited a female predominance of 83.2%, a pattern consistently observed in most studies on the subject [10,12,25,26].

Gender has been reported to significantly influence the preferences for certain nursing specialties [10,25,27,28], although some studies have not found statistically significant differences in this regard [29]. The results of our study align with the findings of Matarese et al. [10] and Hsu et al. [26], showing a clear preference among female students for Paediatrics and Obstetrics and Gynaecology (Midwifery), while male students exhibited a stronger preference for Emergency Medicine. Conversely, certain areas, such as Geriatrics and Mental Health and Psychiatric Nursing, were only marginally preferred by both genders, corroborating the findings from other studies [10,12,21,26,30].

Historically, there has been a gender bias in the selection of nursing as a profession, with societal expectations, gender discrimination, and the socialization of women influencing their career choices over other professions [27,31,32,33,34]. Additionally, female nursing students may feel more inclined to choose certain specialties over others based on their comfort level in environments that are predominantly male. This gender bias is particularly pronounced in areas such as maternal nursing–midwifery [35,36,37]. Therefore, it is crucial to implement policies in nursing education aimed at reducing gender bias and mitigating its impact on nursing students’ specialization choices during their studies.

The areas of greatest preference among students were Emergency Nursing and General Nursing. These findings are consistent with other studies, particularly concerning the preference for Emergency Nursing [10,11,21]. Intensive and Critical Care Nursing also garnered a notable level of preference among the students in our study. However, the area of Operating Theatre and Anaesthesia Nursing received relatively lower interest, contrasting with the results reported in other studies [10,12,30].

Principal component factor analysis, an accepted method in the social sciences for establishing the relationships between areas, was used to establish the number of clusters [38]. This analysis revealed a first distinct cluster comprising Intensive and Critical Care Nursing, Emergency Nursing and Operating Theatre and Anaesthesia Nursing, which can be considered as ‘high-tech’ areas, traditionally among those preferred by nursing students [10,11,29,30]. The second cluster, which included Obstetric Gynaecological Nursing–Midwifery, Paediatric Nursing and Mental Health and Psychiatric Nursing, corresponds to specialities referring to very specific populations (pregnant women, children, persons with mental problems) and coincides with well-established nursing specialities in Spain [7]. Finally, the third cluster included areas that could be considered to affect more general populations and perhaps have a community dimension.

Several explanations have been proposed to account for the attraction of nursing students to ‘high-tech and complex’ areas. These range from the sheer fascination with cutting-edge technologies [39], to the influence of numerous films and television series that dramatize healthcare settings, particularly in high-tech areas such as emergency rooms, rescue services, and operating rooms. For instance, García and Sievering [40] pointed out that medical dramas on television can shape students’ career aspirations, potentially leading to misconceptions about the healthcare profession, which may be further distorted by social media [40]. Although the specific influence of these television series on nursing students has been minimally studied, it cannot be entirely dismissed. Interestingly, despite the high preference for these highly technical areas among students, numerous studies report high levels of job dissatisfaction, burnout, anxiety, and depression among the nurses working in these environments [41,42,43,44]. This dissonance between the aspirations of students and the realities which they face in their professional careers is particularly noteworthy.

This preference may negatively impact other fields, such as community nursing, which has traditionally been regarded as an area of low preference among nursing students [16,29,45,46]. However, in our study, community nursing was ranked as the third-most popular area. Similar to the high-tech specialties, the perceptions of the role of the community nurse are heavily influenced by media portrayals and social stereotypes, leading to an ambiguous and complex view of family and community nursing among students, as reported in several studies [47,48].

The preference for Paediatrics as a chosen specialty in our study was lower compared to other studies [8,10], yet similar to findings from previous research conducted in a similar context [21]. Statistical analysis in our study revealed a significant relationship between gender and the choice of Paediatrics (*p* = 0.020), possibly influenced by traditional gender roles or social stereotypes that perceive this specialty as more feminine and emotionally demanding. Additionally, a strong positive correlation was found between the preference for Paediatric Nursing and Obstetric-Gynaecological Nursing (Midwifery) (r = 0.435), forming a cluster that also included Mental Health and Psychiatric Nursing. The most influential factor in the choice of Paediatric Nursing in our study was having children (*p* = 0.004, effect size = 0.425). It has been reported that nurses with children are more susceptible to compassion fatigue [49], which may be related; it is plausible that nursing students with children may not feel psychologically prepared to handle the emotionally challenging situations often encountered in Paediatric Nursing practice [49,50,51].

It is important to highlight that the two areas of care that garnered the least interest among the students were Geriatric Nursing and Mental Health and Psychiatric Nursing. These findings are consistent with the results of several other studies [10,11,12,21,26,30,52,53]. The perception of Geriatric Nursing as an area with low professional status, high workload, and a lack of positive experiences in caring for the elderly has been identified as key factors contributing to this low preference among nursing students [53,54].

Similarly, Mental Health and Psychiatric Nursing received low scores, making it the least preferred area, excluding the category “Other areas (teaching, management, research)”, which is better classified as a miscellaneous category. The low appeal of Mental Health and Psychiatric Nursing to nursing students has been consistently reported in the literature [10,21,26,28,52,55]. Studies suggest that student nurses may attach some stigma to individuals with mental illness, which poses a significant barrier to choosing to work in this field [56,57].

This study has highlighted a concerning lack of interest among nursing students in certain nursing specialties, which does not align with the current and future needs of the healthcare workforce. Currently, there is a high demand for nurses in both Geriatric Nursing and Mental Health Nursing, and this demand is expected to increase. The significant rise in global life expectancy is associated with a growing need for specialized nurse care for the elderly [54,58,59]. The lack of interest among potential nurses in geriatric care poses serious challenges for healthcare organizations. Similarly, the COVID-19 pandemic has had a profound impact on the mental health of the population [60,61,62]. This surge in mental health issues underscores the critical need for mental health nurses, whose expertise is more essential than ever.

It is important to emphasize that students’ future career intentions may be significantly influenced by their experiences during clinical rotations [8,10,11,12,28,30]. Nursing is a profession with a highly practical character, making clinical rotations crucial not only for providing students with adequate training but also for influencing their future preferences and their commitment to successfully completing their studies. It is essential that students are well prepared to manage and navigate the challenges which they encounter during clinical rotations, as this can enhance their overall experience and prevent potential negative outcomes that might lead to dropout or the stigmatization of certain placement areas [8]. The possible influence that nursing instructors may have on students during these rotations also remains to be studied.

Furthermore, careful planning of students’ exposure to various areas and specialties is necessary, following well-defined criteria aimed at improving the engagement in those areas where the preferences are typically lower. This approach can enhance the learning opportunities and facilitate recruitment across all nursing specialties [13]. A limited availability of placements in certain nursing specialties at some educational institutions is a significant issue that may contribute to the lack of exposure to these areas and warrants greater attention.

In Spain, the access to some nursing specialties is regulated by a national speciality programme that includes a residency programme [7,63]. Among the ten areas offered to students in this study, only five are currently recognized as specialties by the Spanish Ministry of Education. The remaining areas require nursing graduates to pursue additional training and professional development through alternative pathways such as master’s degrees, university certifications, or continuing education programmes. As previously mentioned, there is a Medical-Surgical Nursing specialty that has yet to be implemented, which theoretically encompasses Emergency Nursing, Intensive and Critical Care, and Operating Theatre Nursing [64,65,66]. It is noteworthy that this specialty, which includes areas of high student preference, as demonstrated in this study, remains unregulated and undeveloped in Spain, in contrast to other less preferred areas, such as Mental Health Nursing and Geriatric Nursing, where fully established specialty programmes exist [65,66]. This disparity represents a critical issue that urgently needs to be addressed in our country [64,66].

The results indicate that 49.2% of students intend to pursue a nursing specialty through the Ministry’s residency programme. However, a higher percentage (70.4%) expressed a preference for postgraduate programmes, such as official master’s degrees, university expert courses, or other forms of postgraduate training. Although Spain offers a wide range of postgraduate education opportunities for nurses, the number of available residency positions for nursing specialties is very limited (only 2171 vacancies in the 2024/2025 application cycle across six specialties) [67]. Given that between 2021 and 2022, a total of 11,166 nurses graduated from Spanish universities, this significant limitation restricts the access for many graduates who wish to pursue a specialized nursing residency [68]. In Spain, the system for regulating the access to certain nursing specialties can be seen in two contrasting lights. On the positive side, this external system ensures a predetermined flow of professionals into specific specialties based on the available vacancies, independent of individual preferences. On the negative side, however, the limited number of residency slots can lead to frustration, a lack of motivation, and even push graduates to leave the profession due to the lack of freedom and opportunity to direct their professional development toward their desired specialty. In our view, it is essential that the number of available positions in nursing specialties be commensurate with the number of graduates. Additionally the development of new roles and specialization pathways in Spain, such as the implementation of advanced practice nursing [69,70,71], should be further developed.

It is important to note that the questionnaire did not include an open-ended option for students to express other professional and/or academic interests (e.g., a career in military health, pursuing another degree). As a result, the full range of students’ professional aspirations may not have been captured. Further studies are needed to gather more comprehensive information on the diverse interests and motivations of nursing students in our setting.

Special attention should be given to the lowest-scoring area: “Other areas (teaching, management, research)”. As noted earlier, this category is somewhat miscellaneous, as it does not represent a specific thematic area but rather encompasses roles that can be undertaken within any of the other nine areas. Only 12 students (6.7%) indicated this area as being highly desirable. Factors such as being male, having prior work experience in healthcare, and intending to pursue a postgraduate or doctoral degree were associated with a greater preference for this area (but the effect sizes were small). The correlations between this area and the other areas were negative in all cases, except for Intensive and Critical Care Nursing (r = 0.016, *p* = 0.828) and Family and Community Nursing (r = 0.178, *p* = 0.017).

Research is an integral part of the nursing profession and is essential for advancing the field [72]. Undergraduate nursing curricula should therefore include content, teaching strategies, and training focused on developing research and evidence-based practice (EBP) skills, enabling nurses to integrate valid and relevant research findings into their practice [73,74]. However, teaching research and EBP to undergraduate nursing students presents significant challenges [75,76]. Some studies have reported that undergraduate students often hold negative attitudes or beliefs towards research [75,76]. While students may not fully grasp the importance of the connection between research and clinical practice, it is crucial to instil this understanding in nursing students [74]. Various programmes have been designed to achieve this, but their effectiveness has varied [73,77,78]. The findings from our study highlight the need to consider implementing strategies or programmes to enhance the appeal of research among nursing students at the ULPGC.

### Limitations

It is necessary to note the limitations of this study. Although efforts were made to recruit a representative sample, the low participation rate (41.62%) limits the generalizability of the findings. A higher participation rate would have provided a more accurate and specific understanding of the perspectives of the students enrolled in this degree programme. Therefore, the results should be interpreted with caution. On the other hand, the potential relationship between students’ preferences and their clinical rotations was not examined, despite existing evidence that such experiences can significantly impact their preferences. The complexity of reliably and systematically collecting data on this variable contributed to the decision not to include it in this study. Furthermore, the preference questionnaire did not include the specialty of Occupational Nursing, primarily due to the lack of exposure that students have to this area during their Nursing Degree.

Finally, the categorization of areas of preference was based on the Spanish health, academic, and regulatory context. While this categorization is largely consistent with those used in most studies on this topic, there may be some differences that should be considered when comparing the results.

## 5. Conclusions

This study aimed to identify the employment preferences of third- and fourth-year Nursing Degree students at the ULPGC. The findings revealed that ULPGC nursing students exhibit strong preferences for certain areas, such as “Emergency Nursing”, “General Nursing”, and “Family and Community Nursing”, while showing low interest in others, including “Other Areas-teaching, management, research”, “Mental Health and Psychiatric Nursing”, and “Geriatric Nursing”. This suggests that the specialization preferences of nursing students do not align with the areas where there is the greatest demand from healthcare institutions. Furthermore, the students demonstrated a tendency to prioritize traditional caregiving roles over roles in research, teaching, or management.

A significant proportion of the students expressed intentions to pursue postgraduate studies, with a slightly smaller percentage interested in the Ministry’s residency programme for nursing specialization (EIR). Only about a quarter of the students indicated plans to pursue a doctoral degree. The study also identified closely correlated areas, suggesting that combined strategies could be employed to enhance the interest in those areas with lower preference. There is a clear need to implement strategies aimed at increasing the attractiveness of research roles to nursing students.

## Figures and Tables

**Table 1:** Frequencies and percentages of the socio-demographic variables considered and differences between the groups according to the campuses to which the participants belonged.

Variable	Total n = 179 (100.0%)	Campus Gran Canaria n = 116 (64.8%)	Campus Fuerteventura n = 37 (20.7%)	Campus Lanzarote n = 26 (14.5%)	
		N (%)	N (%)	N (%)	*p* value ^2^
Gender ^1^					*p* = 0.649
Women	149 (83.2%)	93 (52.0%)	33 (18.4%)	23 (12.8%)	
Men	29 (16.2%)	22 (12.3%)	4 (2.2%)	3 (1.7%)	
Nationality					*p* = 0.485
Spanish	176 (98.3%)	115 (64.2%)	36 (20.1%)	125 (14.0%)	
Other nationality	3 (1.7%)	1 (0.6%)	1 (0.6%)	1 (0.6%)	
Children					*p* = 0.180
Yes	16 (8.9%)	9 (5.0%)	6 (3.4%)	1 (0.6%)	
No	163 (91.1%)	107 (59.8%)	31 (17.3%)	25 (14%)	
Academic year					*p* = 0.752
3rd year	81 (45.3%)	54 (30.2%)	17 (9.5%)	10 (5.6%)	
4th year	98 (54.7%)	62 (34.6%)	20 (11.2%)	16 (8.9%)	
Marital status					*p* = 0.010 *
Single	160 (89.4%)	104 (58.1%)	31 (17.3%)	25 (14.0%)	
Married	12 (6.7%)	10 (5.6%)	1 (0.6%)	1 (0.6%)	
Divorced	7 (3.9%)	2 (1.1%)	5 (2.8%)	0 (0.0%)	
Previous health work experience					*p* = 0.030 *
Yes	32 (18.0%)	20 (11.2%)	11 (6.2%)	1 (0.6%)	
No	146 (82%)	95 (53.4%)	26 (14.6%)	25 (14.0%)	
Intention to carry out a nursing speciality after completing the degree studies					*p* = 0.295
Yes	88 (49.2%)	62 (34.6%)	15 (8.4%)	11 (6.1%)	
No	91 (50.8%)	54 (30.2%)	22 (12.3%)	15 (8.4%)	
Intention to carry out postgraduate studies after completion of degree studies					*p* = 0.002 *
Yes	126 (70.4%)	76 (42.5%)	31 (17.3%)	19 (10.6%)	
No	53 (29.6%)	40 (22.3%)	6 (3.4%)	7 (3.9%)	
Intention to carry out doctoral programmes after completing degree studies					*p* = 0.586
Yes	48 (26.8%)	34 (19%)	9 (5.0%)	5 (2.8%)	
No	131 (73.2%)	82 (45.8%)	28 (15.6%)	21 (11.7%)	
Age ^3^	M (SD)	M (SD)	M (SD)	M (SD)	*p* = 0.026 ^4^
	25.2 (8.91)	24.8 (8.30)	27.8 (11.52)	23.1 (6.50)	

^1^ For the gender variable, non-binary gender (n = 178) was excluded from the bivariate analysis; ^2^ *p*-value obtained with chi-square. * Statistically significant *p*-value; ^3^ expressed in means and standard deviations; ^4^ *p*-value obtained with Kruskall–Wallis.

**Table 2:** Statistics for all the thematic areas.

Thematic Areas ^1^	M [CI 95%] ^2^	SD ^3^	% of Agreement ^4^	Floor Not at All Desired ^5^ N (%)	Ceiling (Upper) Very Much Desired ^5^ N (%)
Emergency Nursing	2.70 [2.54–2.87]	1.13	29.8%	8 (4.5%)	51 (28.5%)
General Nursing	2.70 [2.56–2.84]	0.96	20.6%	4 (2.2%)	36 (20.1%)
Family and Community Nursing—Primary Care	2.51 [2.36–2.67]	1.05	19.4%	9 (5.0%)	33 (18.4%)
Intensive and Critical Care Nursing	2.37 [2.21–2.53]	1.08	17.3%	11 (6.1%)	29 (16.2%)
Obstetric-Gynaecological Nursing (Midwifery)	2.05 [1.85–2.25]	1.33	22.4%	27 (15.1%)	34 (19.0%)
Operating Theatre and Anaesthesia Nursing	1.98 [1.81–2.15]	1.14	10.1%	21 (11.7%)	16 (8.9%)
Paediatric Nursing	1.88 [1.70–2.05]	1.20	10.6%	28 (15.6%)	16 (8.9%)
Geriatric Nursing	1.83 [1.65–2.00]	1.20	10.7%	29 (16.2%)	16 (8.9%)
Mental Health and Psychiatric Nursing	1.78 [1.60–1.95]	1.20	8.2%	33 (18.4%)	12 (6.7%)
Others areas (teaching, management, research)	1.43 [1.24–1.64]	1.26	9.7%	55 (30.7%)	12 (6.7%)

^1^ Thematic areas in order of highest to lowest preference; ^2^ means and 95% confidence intervals; ^3^ standard deviation; ^4^ percentage of agreement calculated as number of students scoring 4 or 5 divided by the total number of students ×100; ^5^ only upper (ceiling) or lower (floor) responses are displayed per item. Ceiling responses refer to “Very much desired” and Floor responses refer to “Not at all desired”.

**Table 3:** Statistics for the bivariate analysis.

Thematic Areas	Paediatric Nursing	Midwifery	Mental Health and Psychiatric Nursing	Emergency Nursing	Operating Theatre and Anaesthesia Nursing	General Nursing	Intensive and Critical Care Nursing	Family and Community Nursing—Primary Care	Geriatric Nursing	Other Areas (Teaching, Management, Research)
Academic year										
Third year (n = 81)	1.88 (1.19)	2.04 (1.30)	1.72 (1.18)	2.86 (1.06)	2.30 (1.08)	2.53 (0.95)	2.38 (0.90)	2.42 (1.04)	1.72 (1.15)	1.40 (1.23)
Fourth year (n = 98)	1.88 (1.21)	2.06 (1.37)	1.83 (1.22)	2.57 (1.18)	1.71 (1.21)	2.84 (0.95)	2.36 (1.21)	2.59 (1.06)	1.92 (1.24)	1.46 (1.29)
*p*-value	0.986	0.927	0.629	0.112	0.001 *	0.026 *	0.801	0.242	0.245	0.780
Effect size	0.002	0.008	0.041	0.133	0.280	0.183	0.021	0.097	0.098	0.024
Gender										
Women (n = 149)	1.95 (1.18)	2.17 (1.32)	1.80 (1.23)	2.62 (1.14)	1.94 (1.76)	2.68 (1.00)	2.31 (1.10)	2.48 (1.03)	1.82 (1.20)	1.30 (1.24)
Men (n = 29)	1.41 (1.15)	1.34 (1.14)	1.69 (1.04)	3.10 (1.01)	2.17 (0.93)	2.72 (0.70)	2.69 (0.93)	2.69 (1.17)	1.90 (1.24)	2.14 (1.13)
*p*-value	0.020 *	0.002 *	0.631	0.032 *	0.329	0.809	0.094	0.234	0.822	0.001 *
Effect size	0.266	0.352	0.054	0.243	0.111	0.027	0.189	0.134	0.026	0.388
Children										
Yes (n = 16)	1.06 (0.93)	2.25 (1.00)	1.88 (1.36)	2.19 (1.42)	1.75 (1.23)	2.69 (1.35)	2.38 (1.26)	2.94 (0.85)	2.44 (1.59)	2.06 (1.48)
No (n = 163)	1.96 (1.19)	2.03 (1.36)	1.77 (1.18)	2.75 (1.09)	2.00 (1.13)	2.70 (0.92)	2.37 (1.07)	2.47 (1.06)	1.77 (1.15)	1.37 (1.22)
*p*-value	0.004 *	0.485	0.664	0.116	0.489	0.578	0.818	0.082	0.057	0.060
Effect size	0.425	0.104	0.064	0.230	0.102	0.080	0.034	0.253	0.281	0.278
Previous health work experience										
Yes (n = 32)	1.47 (1.22)	2.09 (1.40)	1.63 (1.31)	2.91 (0.86)	2.06 (1.08)	2.84 (0.77)	2.56 (0.95)	2.53 (1.16)	2.13 (1.21)	1.94 (1.24)
No (n = 146)	1.97 (1.18)	2.04 (1.33)	1.82 (1.17)	2.66 (1.18)	1.95 (1.15)	2.66 (1.00)	2.34 (1.10)	2.51 (1.03)	1.76 (1.20)	1.33 (1.24)
*p*-value	0.031 *	0.807	0.464	0.443	0.521	0.467	0.281	0.758	0.113	0.011 *
Effect size	0.237	0.027	0.081	0.084	0.070	0.078	0.117	0.034	0.174	0.280
Intention to carry out a nursing speciality after completing the degree studies										
Yes (n = 88)	2.31 (1.18)	2.51 (1.43)	1.83 (1.24)	2.57 (1.15)	1.82 (1.17)	2.50 (1.02)	2.17 (1.02)	2.64 (1.08)	1.70 (1.22)	1.42 (1.25)
No (n = 91)	1.46 (1.07)	1.60 (1.06)	1.73 (1.16)	2.84 (1.10)	2.13 (1.09)	2.89 (0.86)	2.56 (1.11)	2.40 (1.01)	1.95 (1.18)	1.44 (1.28)
*p*-value	0.001 *	0.001 *	0.590	0.126	0.089	0.009 *	0.012 *	0.116	0.161	0.987
Effect size	0.403	0.381	0.045	0.128	0.143	0.216	0.209	0.130	0.118	0.002
Intention to carry out postgraduate studies after the completion of degree studies										
Yes (n = 126)	1.87 (1.21)	2.10 (1.30)	1.70 (1.24)	2.80 (1.12)	2.09 (1.12)	2.68 (0.99)	2.50 (1.70)	2.43 (1.04)	1.75 (1.20)	1.59 (1.25)
No (n = 53)	1.89 (1.19)	1.94 (1.42)	1.96 (1.09)	2.47 (1.12)	1.72 (1.15)	2.74 (0.90)	2.06 (1.04)	2.72 (1.06)	2.02 (1.20)	1.06 (1.20)
*p*-value	0.943	0.499	0.186	0.049 *	0.068	0.800	0.011 *	0.062	0.145	0.007 *
Effect size	0.007	0.063	0.122	0.180	0.168	0.023	0.232	0.170	0.134	0.247
Intention to carry out doctoral programmes after completing degree studies										
Yes (n = 48)	1.92 (1.27)	2.13 (1.32)	1.88 (1.23)	2.96 (1.15)	2.27(1.14)	2.73 (0.89)	2.73 (1.13)	2.42 (1.22)	1.67 (1.26)	1.92 (1.33)
No (n = 131)	1.86 (1.18)	2.02 (1.34)	1.74 (1.19)	2.61 (1.11)	1.87 (1.12)	2.69 (0.99)	2.24 (1.04)	2.55 (0.99)	1.89 (1.18)	1.25 (1.19)
*p*-value	0.963	0.625	0.497	0.035 *	0.037 *	1.000	0.004 *	0.684	0.264	0.003 *
Effect size	0.005	0.047	0.065	0.199	0.198	0.000	0.271	0.038	0.106	0.285

* Statistically significant *p*-value.

**Table 4:** Correlation matrix for the preference of thematic areas.

		Paediatric Nursing	Midwifery	Mental Health and Psychiatric Nursing	Emergency Nursing	Operating Theatre and Anaesthesia Nursing	General Nursing	Intensive and Critical Care Nursing	Family and Community Nursing—Primary Care	Geriatric Nursing	Other Areas (Teaching, Management, Research)
Paediatric Nursing	Rho Spearman	------------									
	*p*-value	----------									
Midwifery	Rho Spearman	0.435	--------								
	*p*-value	<0.001 *	--------								
Mental Health and Psychiatric Nursing	Rho Spearman	0.025	0.132	---------							
	*p*-value	0.739	0.077	----------							
Emergency Nursing	Rho Spearman	−0.066	−0.183	−0.241	------------						
	*p*-value	0.382	0.014 *	0.001 *	------------						
Operating Theatre and Anaesthesia Nursing	Rho Spearman	−0.050	−0.066	−0.195	0.370	-------------					
	*p*-value	0.508	0.383	0.009	<0.001 *	--------------					
General Nursing	Rho Spearman	−0.006	−0.172	−0.160	0.098	0.042	------------				
	*p*-value	0.940	0.021 *	0.032 *	0.192	0.577	------------				
Intensive and Critical Care Nursing	Rho Spearman	−0.016	−0.141	−0.258	0.510	0.339	0.203	--------------			
	*p*-value	0.827	0.061	<0.001 *	<0.001 *	<0.001 *	0.006 *	--------------			
Family and Community Nursing—Primary Care	Rho Spearman	0.002	0.085	0.019	−0.162	−0.234	−0.049	−0.177	-------------		
	*p*-value	0.984	0.256	0.802	0.030 *	0.002 *	0.515	0.018 *	--------------		
Geriatric Nursing	Rho Spearman	−0.150	−0.182	0.114	−0.110	−0.174	0.312	−0.011	0.202	-----------	
	*p*-value	0.045 *	0.015 *	0.129	0.143	0.020 *	<0.001 *	0.888	0.007 *	-----------	
Other areas (teaching, management, research)	Rho Spearman	−0.112	−0.126	−0.003	−0.044	−0.096	−0.079	0.016	0.178	−0.038	------------------
	*p*-value	0.135	0.092	0.9368	0.562	0.199	0.293	0.828	0.017 *	0.609	------------------

Positive moderate correlations between 0.5 and 1/Negative moderate correlations between −0.5 and −1 *p*-value obtained with Rho of Spearman. * Statistically significant *p*-value.

## Data Availability

The data used in this research are confidential and are protected in a coded and anonymized database kept by the research group in accordance with Spanish regulations. However, raw data concerning the preference scores for the thematic areas can be shared with those researchers who contact the corresponding author if requested with a reasoned and logical request.

## Supplementary Materials

The following supporting information can be downloaded at https://www.mdpi.com/article/10.3390/nursrep14040241/s1, Table S1: Checklist STROBE for this study; Table S2: Operational definitions of areas; Table S3: Means and Standard Deviations by Campus for each area, along with *p*-values and effect sizes; Table S4: Means and Standard Deviations by Marital Status for each area, along with *p*-values and effect sizes.

## Public Involvement Statement

There was no public involvement in any aspect of this research.

## Guidelines and Standards Statement

This manuscript was drafted in line with the STROBE guidelines for a cross-sectional study and descriptive research.
