# Supplementary material for: Academic and Employment Preferences of Nursing Students at the University of Las Palmas of Gran Canaria: A Cross-Sectional Study
Source: Nurs Rep. 2024 Nov 1;14(4):3328–45. doi: 10.3390/nursrep14040241 (PMC11587454; doi:10.3390/nursrep14040241)
Supplement: Supplementary file 1 [file nursrep-14-00241-s001.zip › Supplementary Table S2.pdf]

Supplementary Table S2. Operational definitions of areas

-Paediatric Nursing: Health care for newborns and children. Paediatric care services in hospitals and primary care.

-Obstetric-Gynaecological Nursing (Midwifery); Health care for women in pregnancy, childbirth, puerperium and climacteric. Delivery room services and maternity and puerperium wards. Primary care midwives.

-Mental Health and Psychiatric Nursing; health care for people with mental disorders and/or affected by psychiatric pathology.

-Emergency Nursing; health care for people in extra-hospital emergency services, including ambulances and other emergency units (helicopters-rescue units).

-Operating Theatre and Anaesthesia Nursing; health care in operating theatres of all surgical specialities.

-General Nursing; Includes all those services of various specialties that require specialised care, usually in hospitals. Example: Hospitalisation Units for Internal Medicine, Traumatology, Surgery, Neumology, Nephrology, Digestive or Oncology.

-Intensive and Critical Care Nursing; Health care for people in intensive care units and/or resuscitation units. Includes interventional cardiology units - cardiac catheterisation.

-Family and Community Nursing-Primary Care; Care of the individual, family and community in Primary Care. Includes health promotion, protection, recovery and rehabilitation, as well as disease prevention. Primary care health centres.

-Geriatric Nursing; Health care and care for people over 65 years of age, whether in hospitals, social health centres or other settings.

-Other areas; areas not related to direct patient care, such as teaching (university or other educational levels), management (public health and non-health administrations) or research.
