# Supplementary material for: Academic and Employment Preferences of Nursing Students at the University of Las Palmas of Gran Canaria: A Cross-Sectional Study
Source: Nurs Rep. 2024 Nov 1;14(4):3328–45. doi: 10.3390/nursrep14040241 (PMC11587454; doi:10.3390/nursrep14040241)
Supplement: Supplementary file 1 [file nursrep-14-00241-s001.zip › TABLE S3 y S4 Preferences.pdf]

Supplementary Table S3. Statistics by Campuses for each area, along with p-values and effect sizes

	Campus Gran Canaria **(a)** (n=116)	Campus Lanzarote **(b)** (n=26)	Campus Fuerteventura **(c)** (n=37)		Kruskall Wallis[2]	Effect Size	Dwass-Steel-Critchlow-Fligne Post Hoc
**Thematic areas**	M (SD)[1]	M (SD)[1]	M (SD)[1]	X^2	p-value	ε^2	
Paediatric Nursing	1.89(1.21)	2.04(1.11)	1.73(1.22)	0.982	0.612	0.005	N.S.[3]
Obstetric-Gynaecological Nursing (Midwifery)	2.08(1.37)	1.92(1.35)	2.05(1.22)	0.316	0.854	0.001	N.S.
Mental Health and Psychiatric Nursing	1.78(1.22)	1.62(0.98)	1.89(1.26)	0.853	0.653	0.004	N.S.
Emergency Nursing	2.64(1.13)	2.50(1.17)	3.05(1.05)	5.558	0.062	0.031	N.S.
Operating Theatre and Anaesthesia Nursing	2.05(1.12)	1.73(0.92)	1.92(1.32)	2.091	0.352	0.011	N.S.
General Nursing	2.64(0.96)	2.77(1.03)	2.84(0.90)	1.083	0.582	0.006	N.S.
Intensive and Critical Care Nursing	2.34(1.01)	2.27(1.04)	2.54(1.30)	2.359	0.307	0.013	N.S.
Family and Community Nursing-Primary Care	2.46(1.03)	2.50(1.03)	2.70(1.13)	1.317	0.518	0.007	N.S.
Geriatric Nursing	1.73(1.16)	2.04(1.46)	1.97(1.14)	1.965	0.374	0.011	N.S.
Others areas (teaching, management, research)	1.47(1.29)	1.04(1.04)	1.57(1.28)	2.783	0.249	0.015	N.S.

1 Mean (Standard deviation)

2 p-value obtained with Kruskall Wallis

3. N.S. (Not stastistically Significant bettween groups a,b c,)/ a= Campus Gran Canaria; b=Campus Lanzarote; c=Campus Fuerteventura

Supplementary Table S4. *Statistics by Marital Status for each area, along with p-values and effect sizes*

	Single **(a)** (n=160)	Married **(b)** (n=12)	Divorced **(c)** (n=7)		Kruskall Wallis [2]	Effect Size	Dwass-Steel-Critchlow-Fligne Post Hoc [3]
**Thematic areas**	M (SD)[1]	M (SD)[1]	M (SD)[1]	X^2	p-value	ε^2	
Paediatric Nursing	1,95(1,19)	1,25(1,29)	1,29(0,756)	5,307	0,070	0,030	N.S.[4]
Obstetric-Gynaecological Nursing (Midwifery)	2,04(1,36)	2,17(1,19)	2,14(0,900)	0,222	0,895	0,001	N.S.
Mental Health and Psychiatric Nursing	1,78(1,17)	1,33(1,37)	2.43(1,27)	3,553	0,169	0,020	N.S.
Emergency Nursing	2.76(1.10)	1.83(1.34)	2.86(1.07)	6.215	0.045*	0.035	a,b (p=0.036)**
Operating Theatre and Anaesthesia Nursing	2.01(1.13)	1.67(0.985)	1.86(1.57)	0.930	0.628	0.005	N.S.
General Nursing	2.70(0.923)	2.58(1.38)	2.86(1.07)	0.292	0.864	0.002	N.S.
Intensive and Critical Care Nursing	2.38(1.06)	2.25(1.14)	2.43(1.51)	0.310	0.856	0.002	N.S.
Family and Community Nursing-Primary Care	2.46(1.06)	2.75(0.965)	3.43(0.535)	7.093	0.029*	0.040	a,c (p=0.028)**
Geriatric Nursing	1.72(1.13)	2.33(1.50)	3.43(1.13)	13.072	0.001*	0.073	a,c (p=0.003)**
Others areas (teaching, management, research)	1.36(1.23)	2.42(1.31)	1.29(1.38)	6.956	0.031*	0.039	a,b (p=0.024)**

1 Mean (Standard deviation)

*2 p-value obtained with Kruskall Wallis *Statistically significant p-value*

*3 p-value obtained with Dwass-Steel-Critchlow-Fligne Post Hoc **Statistically significant p-value*
a= Single; b=Married; c=Divorced

4 N.S. (Not Statistically Significant between groups a, b, c)
